# Supplementary material for: Molecular basis for glycan recognition and reaction priming of eukaryotic oligosaccharyltransferase
Source: Nat Commun. 2022 Nov 26;13:7296. doi: 10.1038/s41467-022-35067-x (PMC9701220; doi:10.1038/s41467-022-35067-x)

SUPPLEMENTARY INFORMATION FOR:

Molecular basis for glycan recognition and reaction priming of eukaryotic oligosaccharyltransferase

Ana S. Ramírez[1], Mario de Capitani[2], Giorgio Pesciullesi[2], Julia Kowal[1], Joël S. Bloch[1], Rossitza N. Irobalieva[1], Markus Aebi[3], Jean-Louis Reymond[2] and Kaspar P. Locher[1]*

[1] Institute of Molecular Biology and Biophysics, Eidgenössische Technische Hochschule (ETH), Zürich, Switzerland.

[2] Department of Chemistry and Biochemistry, University of Bern, Bern, Switzerland

[3] Institute of Microbiology, Eidgenössische Technische Hochschule (ETH), Zürich, Switzerland.

*Correspondence to: locher@mol.biol.ethz.ch

This file contains:

Supplementary Figures 1 to 10

Supplementary Table 1

Uncropped gel from Figure 1b

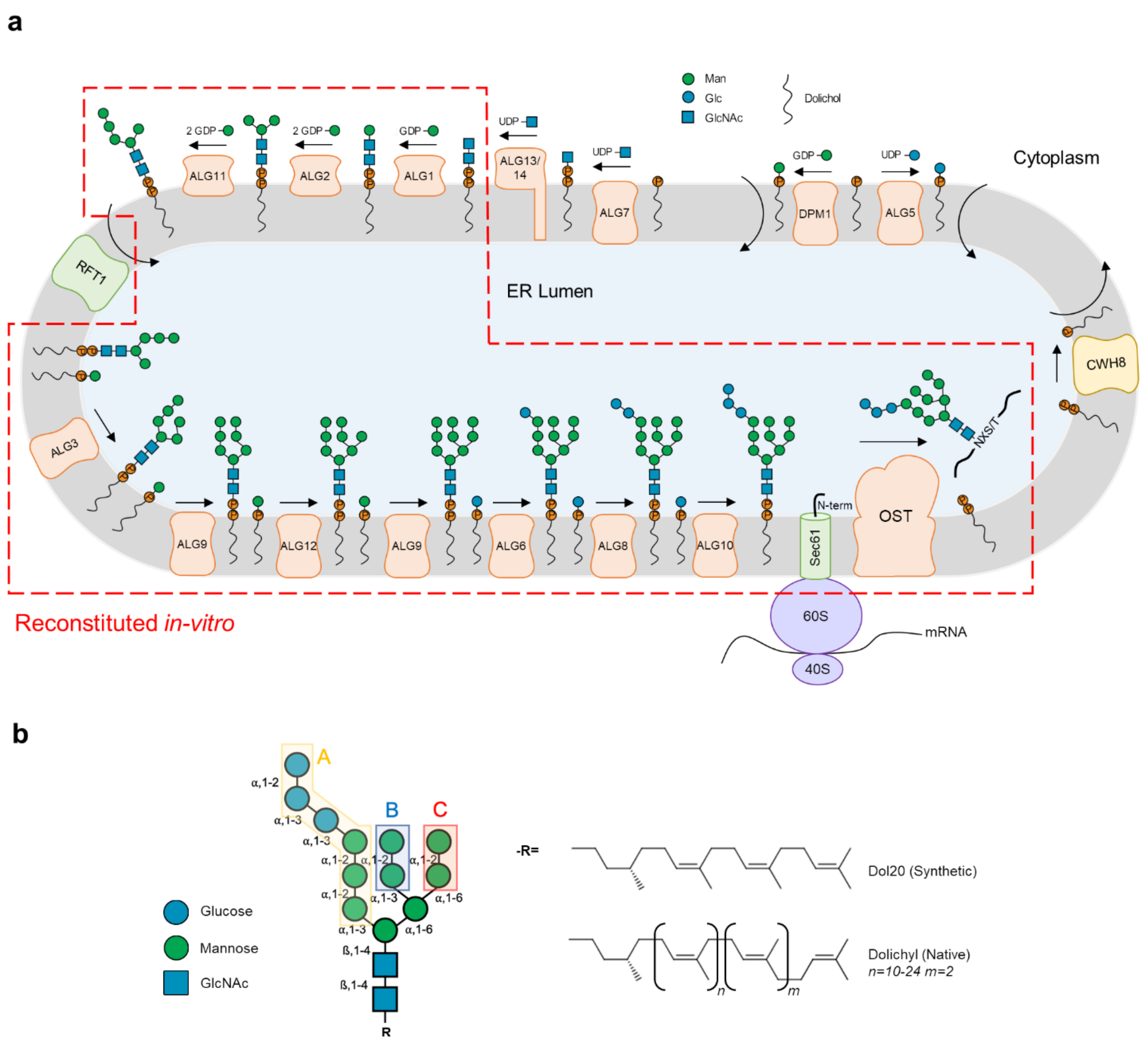

Supplementary Figure 1: Structure of eukaryotic lipid-linked-oligosaccharide.
a. Schematic of the eukaryotic N-glycosylation pathway. Enzyme names are indicated for each reaction. The reactions reconstituted *in vitro* in this study are framed by a dashed red line. **b.** Schematic of the structure of DolPP-GlcNAc$_2$Man$_9$Glc$_3$. The three branches A, B and C are shown and labeled. Glycosidic linkages are indicated for each bond. The chemical structure of the native dolichyl tail and the synthetic Dol20 are shown.

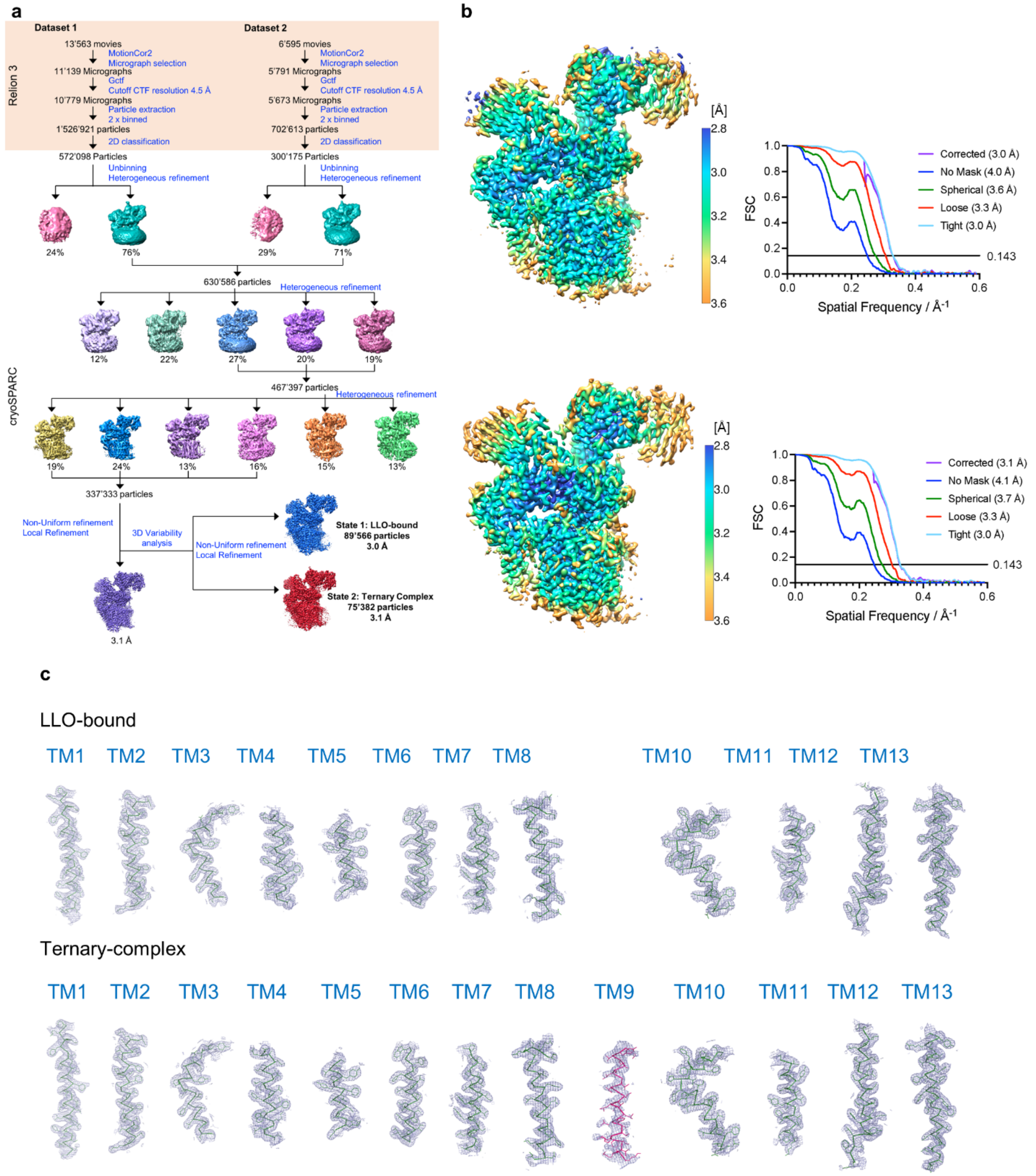

Supplementary Figure 2: Cryo-EM data processing of the ternary-complex dataset.
a. Pipeline of the EM data processing of the ternary-complex dataset, yielding two distinct states: LLO-bound and ternary-complex. **b.** Local resolution map and Fourier shell correlation (FSC) curves for the LLO-bound state (top) and ternary complex (bottom). **c.** Fitting of the TM helices of STT3 in the EM density maps for LLO-bound state (top) and ternary complex (bottom). A region of up to 3 Å around the atoms is shown. TM helices and are shown as sticks. Density is shown as a light blue mesh.

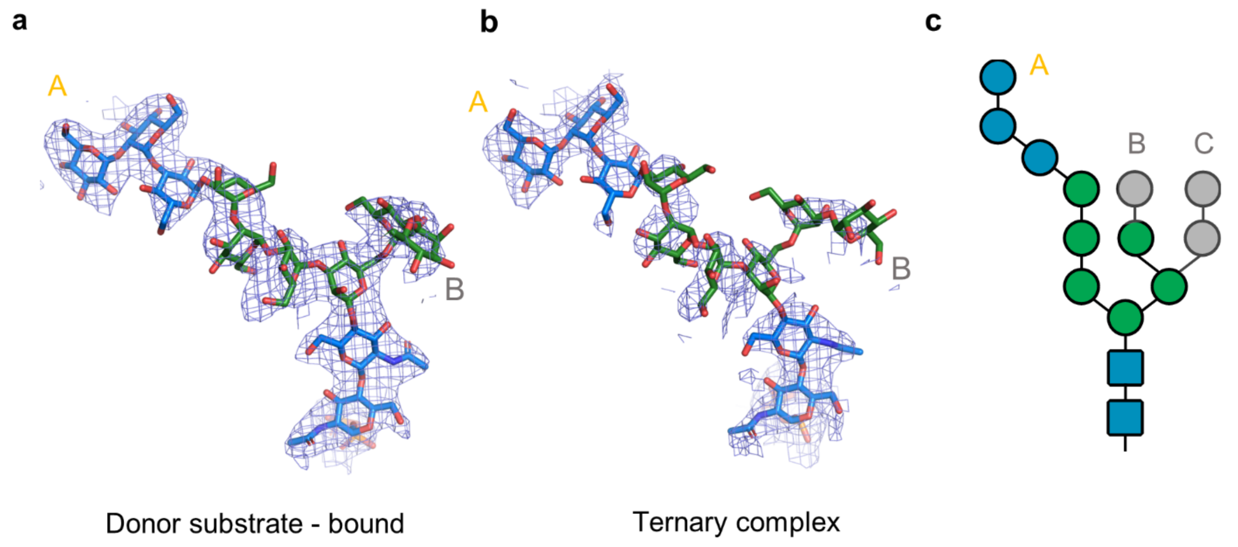

Supplementary Figure 3: EM densities for the glycan moiety of the LLO.
a. EM-density for the LLO glycan in the LLO-bound state. LLO glycan is shown in stick representation with the glucose and N-acetylglucosamine units colored blue and mannose units colored green. The EM density map is shown as a blue mesh. **b.** EM-density for the LLO glycan in the ternary-complex state. The LLO glycan and EM map are shown as in **a**. **c.** Schematic of the glycan moiety of the LLO. Sugar units that were not visible in the EM-maps are colored gray

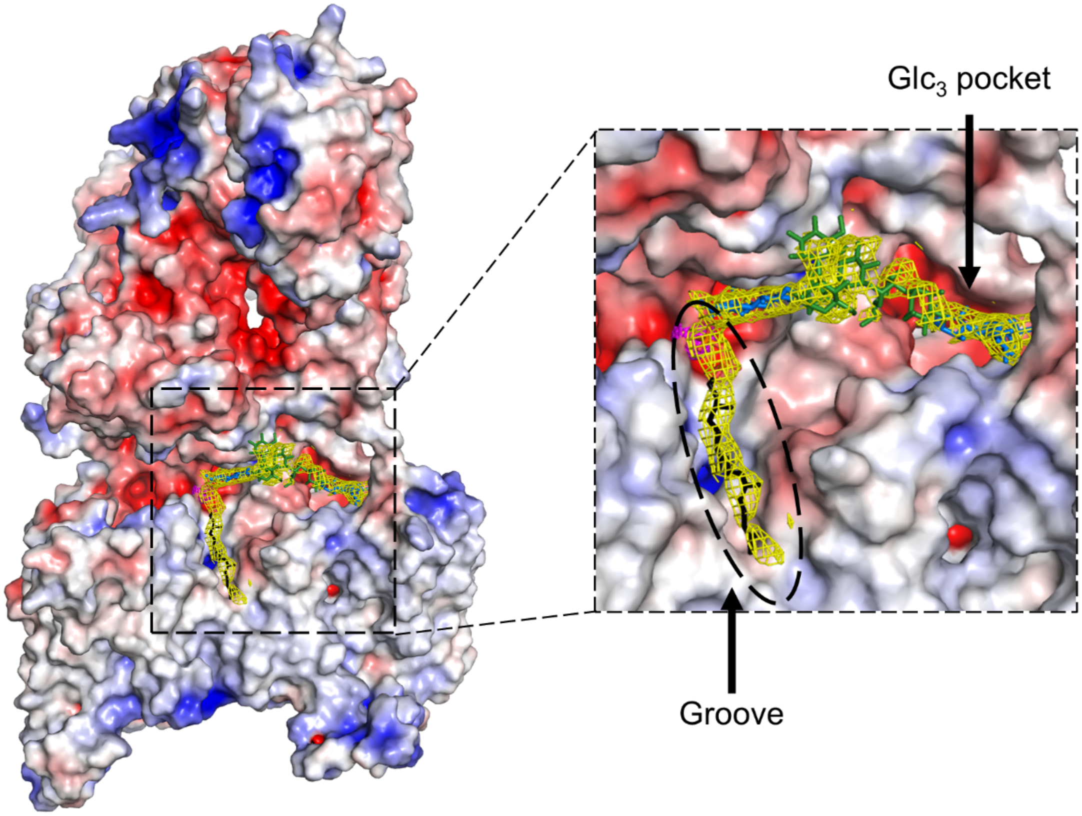

Supplementary Figure 4: Binding of LLO to OST
APBS-calculated electrostatic surface potential of OST, color coded from blue (most positive) to red (most negative) to white (uncharged). Bound LLO is shown as sticks. Dol20 colored black, glucose and N-acetylglucosamine units colored blue and mannose units colored green. Inset shows a close-up view of the binding site for the donor substrate. Glc_3 binding pocket and the hydrophobic groove for the binding of Dol20 are shown and labeled. The EM density map is shown as a yellow mesh.

LLO-bound

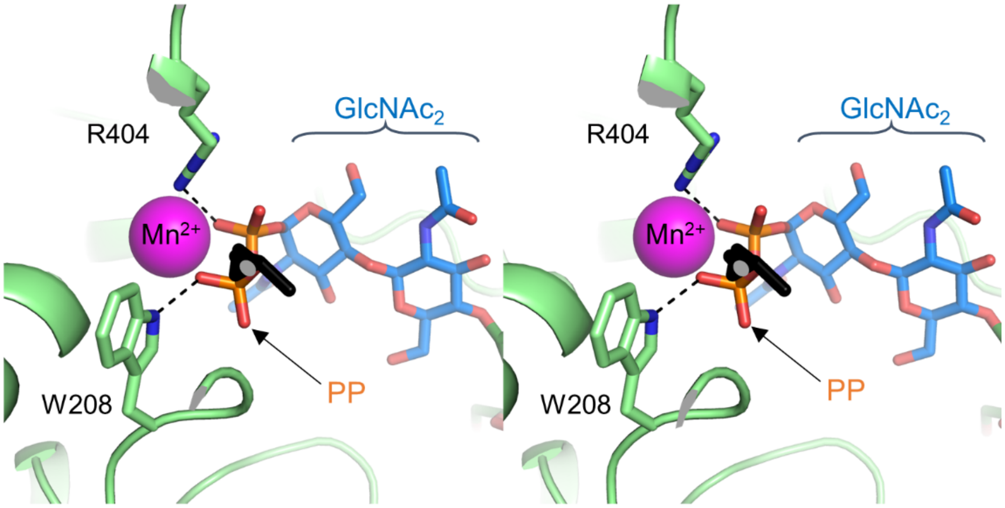

Ternary Complex

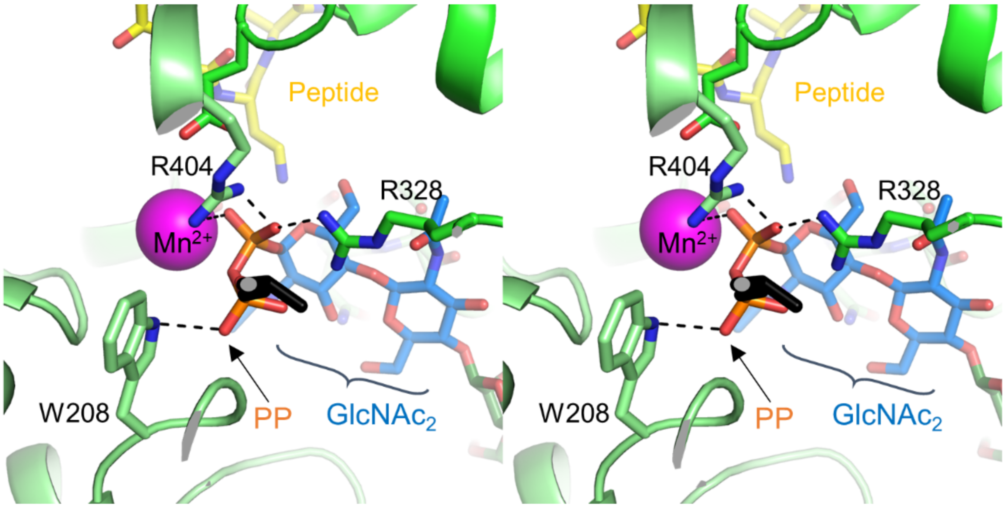

Supplementary Figure 5: Coordination of the pyrophosphate group in the LLO- bound and ternary complex states of yeast OST

Wall-eyed stereo view of the binding site of LLO in the donor substrate-bound (top) and ternary complex states (bottom). The bound LLO is shown as sticks, with the Dol20 moiety colored black and the N-acetylglucosamine units colored blue. The pyrophosphate (PP) group is indicated with a black arrow and labeled. STT3 residues interacting with the pyrophosphate group are shown as sticks and labeled.

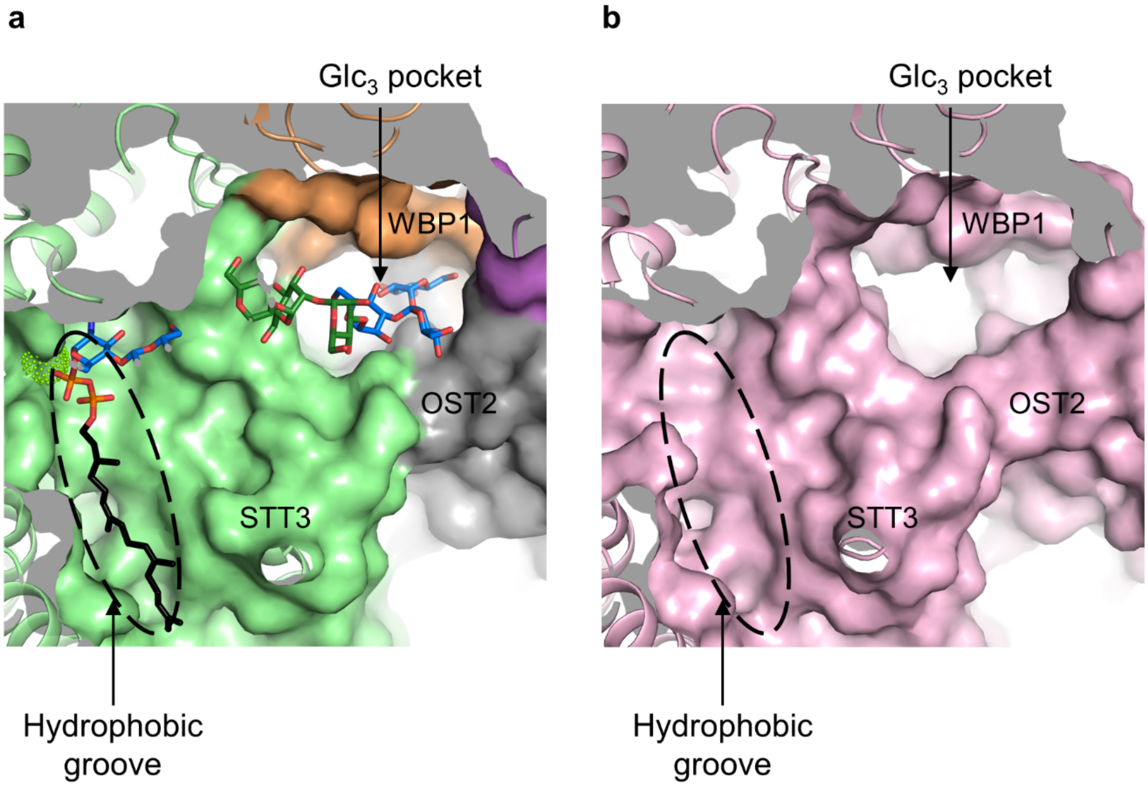

Supplementary Figure 6: Shape complementarity between the LLO binding regions and OST

a. Surface representation of the LLO-bound state of OST. Subunits are colored individually and labeled. The bound LLO is shown as sticks, with the Dol20 moiety colored black, glucose and N-acetylglucosamine units colored blue and mannose units colored green. The hydrophobic groove where the dolichyl tail binds is indicated with a dashed oval. The binding pocket for the terminal Glc_3 moiety of the LLO is indicated and labeled. **b.** Surface representation of the Apo- state of OST (PDB: 6EZN). OST is colored in light pink. The hydrophobic groove for binding of the dolichyl tail and the Glc_3 binding pocket are pre-formed in the absence of substrate bound. Both LLO-binding regions are indicated with black arrows and labeled.

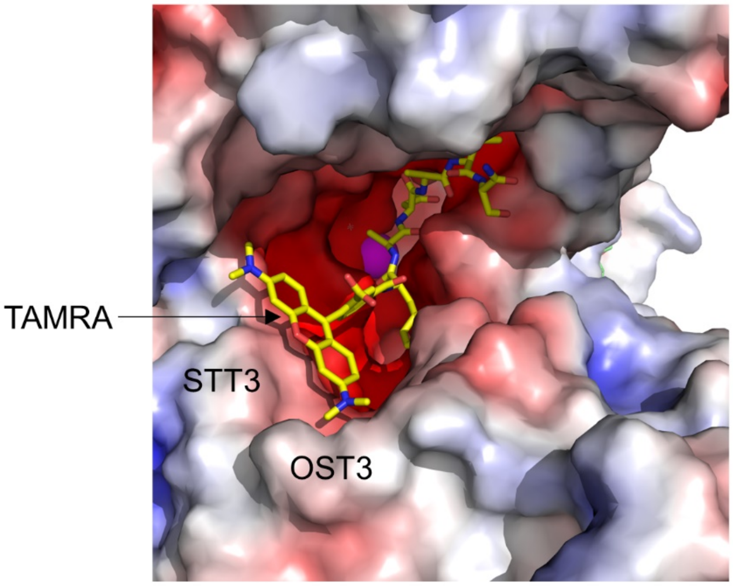

Supplementary Figure 7: Binding of the TAMRA group in the ternary complex
APBS-calculated electrostatic surface potential of OST, color coded from blue (most positive) to red (most negative) to white (uncharged). Bound peptide is shown as yellow sticks. The TAMRA group is shown and labeled. The TAMRA group is accommodated in a binding pocket formed by the subunits STT3 and OST3, which are labeled on the surface representation.

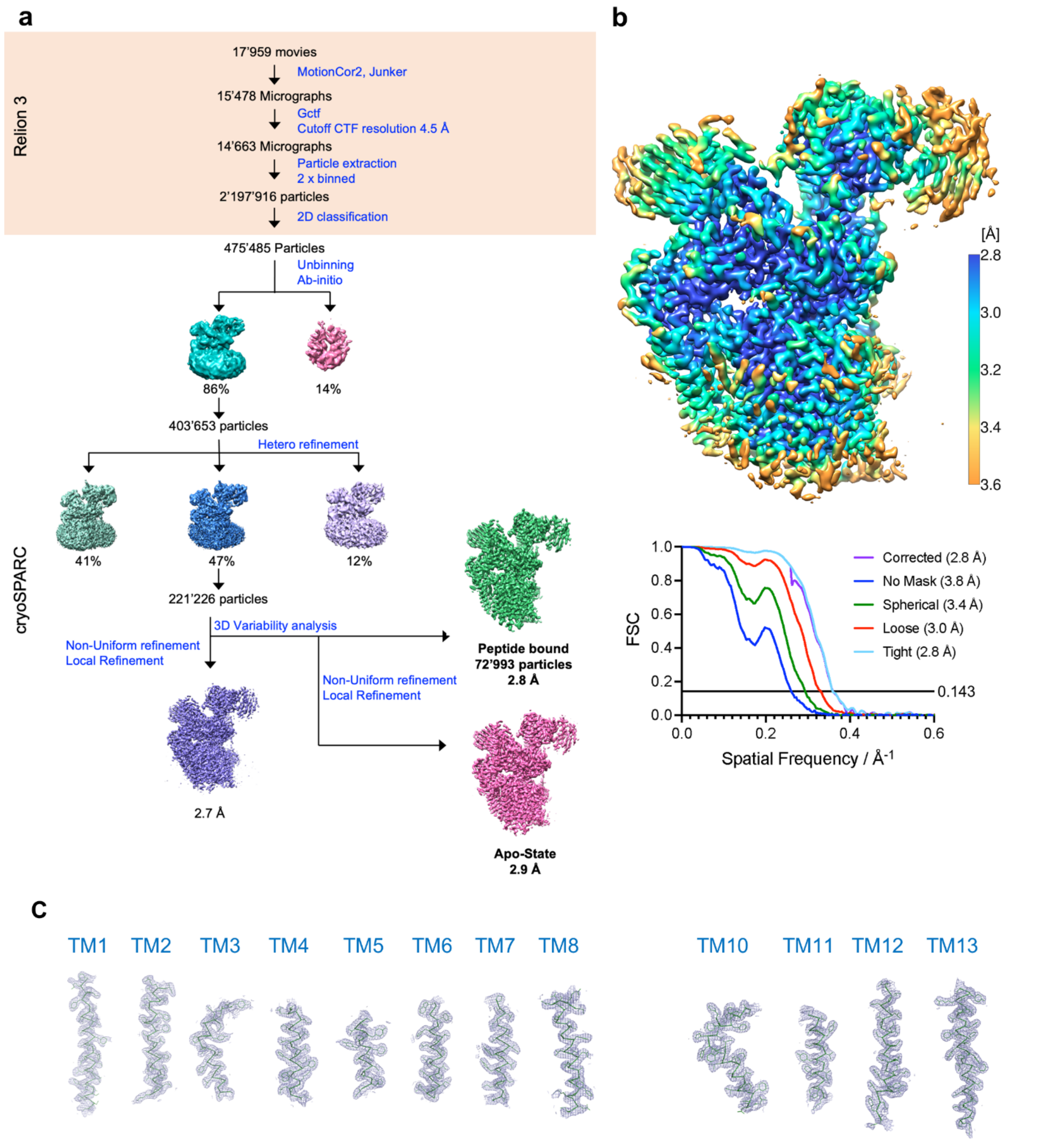

Supplementary Figure 8: Cryo-EM data processing of the peptide-bound dataset.
a. Pipeline of the EM data processing, yielding two distinct states: peptide-bound and Apo-state. **b.** Local resolution map and Fourier shell correlation (FSC) curves for the peptide-bound state. **c.** Fitting of the TM helices of STT3 in the EM density map. A region of up to 3 Å around the atoms is shown. TM helices and are shown as sticks. Density is shown as a light blue mesh.

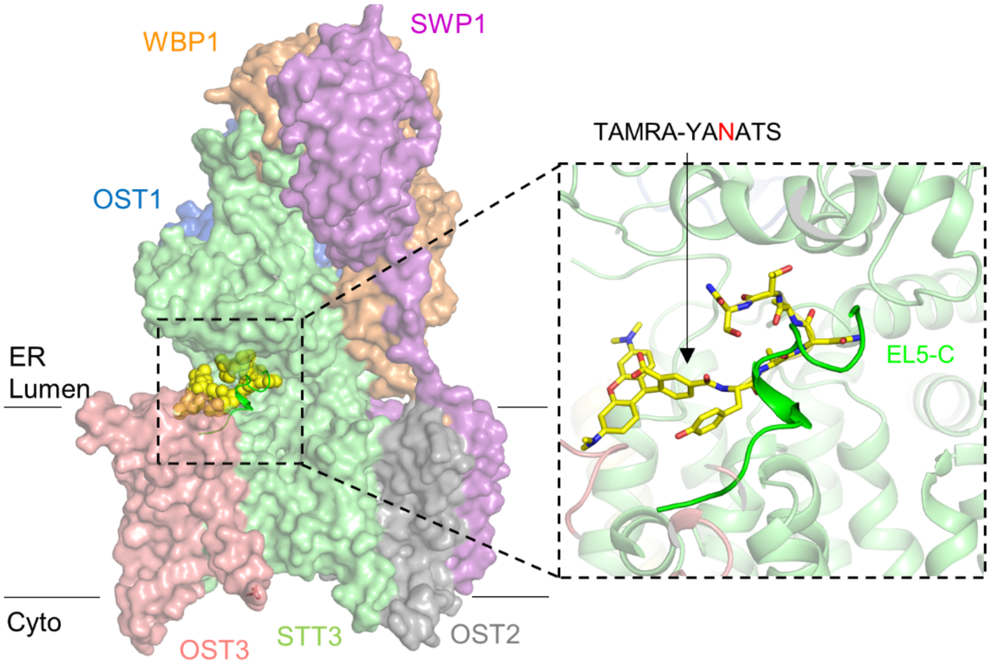

Supplementary Figure 9: Structure of peptide-bound OST
OST is shown in surface representation. Subunits are colored individually and labeled. The ordered C-terminal half of EL5 (EL5-C) is shown as cartoon. Bound peptide is shown as yellow spheres. Inset shows a close-up view of the binding site for the acceptor peptide with OST shown as cartoon and subunits colored as in the surface representation. The acceptor peptide TAMRA-YANATS is shown as yellow sticks and indicated with a black arrow. EL5 is shown as cartoon and colored green.

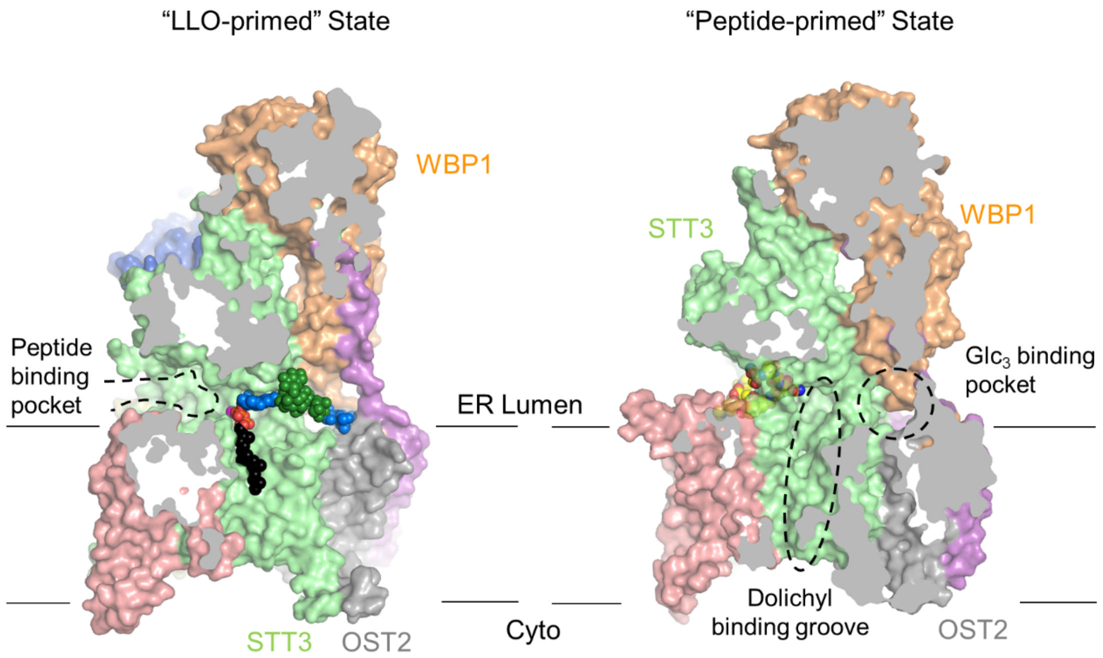

Supplementary Figure 10: Primed states of OST allow binding of the second substrate
Vertical slice through a surface representation of the LLO-primed (left) and peptide-primed (right) states of OST. Subunits involved in substrate binding are individually colored and labelled. In the LLO-primed state (left), the peptide binding pocket remains accessible and it is indicated with a dashed line and labeled. In the peptide-primed state (right), the dolichyl binding groove and the Glc_3 binding pocket remain accessible and are indicated with dashed circles and labeled.

Supplementary Table 1: Cryo-electron microscopy data collection, refinement and validation

	LLO-primed (EMD-15419, PDB 8AGB)	Ternary complex (EMD-15420, PDB 8AGC)	Peptide-primed (EMD-15421, PDB 8AGE)
**Data Collection and Processing**			
Magnification (Nominal)	165,000		130,000
Voltage (kV)	300		300
Electron Exposure ($e^-/Å^2$)	64.0		48.4
Defocus range (µm)			
Pixel size (Å)	0.84		0.66
Symmetry imposed	C1		C1
Initial particle images (no.)	2,229,534		2,197,926
Final particle images (no.)	89,566	75,382	72,993
Map Resolution (Å)	3.0	3.1	2.8
FSC threshold	0.143	0.143	0.143
Map Resolution range	3.0 – 20.0	3.1 – 20.0	2.8 – 20.0
**Model Refinement and validation**			
Initial model used (PDB code)	6EZN	6EZN	6EZN
Model Resolution (Å)	3.1	3.2	2.9
FSC Threshold	0.5	0.5	0.5
Map sharpening *B* factor ($Å^2$)	-81.0	-76.8	-74.4
Model Composition			
Non-hydrogen atoms	17217	17770	17540
Protein residues	2075	2153	2102
Ligands	34	33	28
B factors ($Å^2$) (min/max/mean)			
Protein	40.9/148.5/69.0	29.0/119.3/54.6	25.1/151.2/65.9
Ligands	50.8/103.2/69.7	35.7/101.8/58.0	36.3/134.5/60.3
R.m.s deviations			
Bond lengths (Å)	0.005	0.008	0.003
Bond angles (°)	0.662	0.840	0.677
Validation			
MolProbity Score	2.72	2.14	2.10
Clashscore	8.63	7.07	6.18
Poor rotamers (%)	5.86	3.53	3.88
Ramachandran Plot			
Favored (%)	94.43	95.25	95.57
Allowed (%)	5.52	4.70	4.34
Disallowed (%)	0.05	0.05	0.10

Unncropped gel Figure 1c

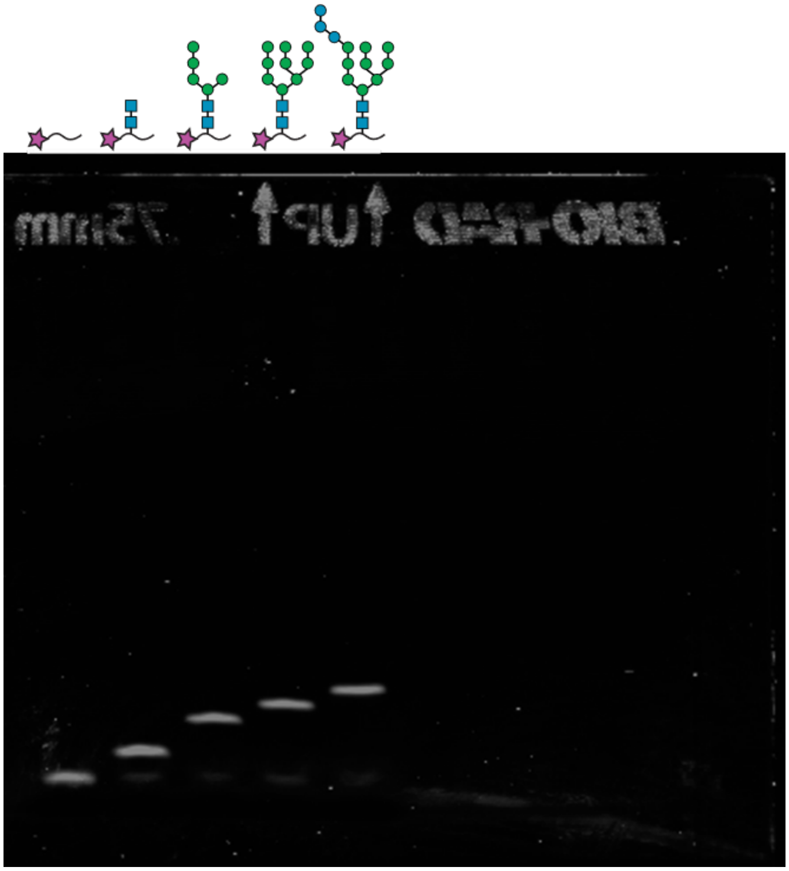

Supplement: Supplementary file 1 — Supplementary Information [file 41467_2022_35067_MOESM1_ESM.pdf]
