# Supplementary material for: Molecular basis for glycan recognition and reaction priming of eukaryotic oligosaccharyltransferase
Source: Nat Commun. 2022 Nov 26;13:7296. doi: 10.1038/s41467-022-35067-x (PMC9701220; doi:10.1038/s41467-022-35067-x)
Supplement: Supplementary file 3 — Reporting Summary [file 41467_2022_35067_MOESM3_ESM.pdf]

nature research

Corresponding author(s): Kaspar P. Locher

Last updated by author(s): Nov 9, 2022

Reporting Summary

Nature Research wishes to improve the reproducibility of the work that we publish. This form provides structure for consistency and transparency in reporting. For further information on Nature Research policies, see Authors & Referees and the Editorial Policy Checklist .

Statistics

For all statistical analyses, confirm that the following items are present in the figure legend, table legend, main text, or Methods section.

n/a	Confirmed	
☐	☒	The exact sample size (*n*) for each experimental group/condition, given as a discrete number and unit of measurement
☒	☐	A statement on whether measurements were taken from distinct samples or whether the same sample was measured repeatedly
☒	☐	The statistical test(s) used AND whether they are one- or two-sided<br>*Only common tests should be described solely by name; describe more complex techniques in the Methods section.*
☒	☐	A description of all covariates tested
☒	☐	A description of any assumptions or corrections, such as tests of normality and adjustment for multiple comparisons
☐	☒	A full description of the statistical parameters including central tendency (e.g. means) or other basic estimates (e.g. regression coefficient) AND variation (e.g. standard deviation) or associated estimates of uncertainty (e.g. confidence intervals)
☒	☐	For null hypothesis testing, the test statistic (e.g. *F*, *t*, *r*) with confidence intervals, effect sizes, degrees of freedom and *P* value noted<br>*Give P values as exact values whenever suitable.*
☒	☐	For Bayesian analysis, information on the choice of priors and Markov chain Monte Carlo settings
☒	☐	For hierarchical and complex designs, identification of the appropriate level for tests and full reporting of outcomes
☒	☐	Estimates of effect sizes (e.g. Cohen's *d*, Pearson's *r*), indicating how they were calculated

Our web collection on statistics for biologists contains articles on many of the points above.

Software and code

Policy information about availability of computer code

Data collection	EPU2 for cryo-EM data collection.
Data analysis	PyMOL 4.6.0, PHENIX 1.17.1, Coot 0.9, Relion 3.1, ChimeraX 0.9, CryoSPARC 3.

For manuscripts utilizing custom algorithms or software that are central to the research but not yet described in published literature, software must be made available to editors/reviewers. We strongly encourage code deposition in a community repository (e.g. GitHub). See the Nature Research guidelines for submitting code & software for further information.

Data

Policy information about availability of data

All manuscripts must include a data availability statement. This statement should provide the following information, where applicable:

- Accession codes, unique identifiers, or web links for publicly available datasets
- A list of figures that have associated raw data
- A description of any restrictions on data availability

Atomic coordinates of the models were deposited in the RCSB Protein Data Bank (PDB) under accession number 8AGB for OST-LLO-primed, 8AGC for OST-ternary-complex, and 8AGE for OST-peptide-primed. The three-dimensional cryo-EM maps were deposited in the Electron Microscopy Data Bank (EMDB) under accession numbers EMD-15419 for OST-LLO-primed, EMD-15420 for OST-ternary-complex and EMD-15421 for OST-peptide-primed. Atomic coordinates of the apo structure of OST under accesion number 6EZN were used as initial mode. Source data are provided with this paper. All other data are available from the corresponding author upon reasonable request.

Field-specific reporting

Please select the one below that is the best fit for your research. If you are not sure, read the appropriate sections before making your selection.

☒ Life sciences ☐ Behavioural & social sciences ☐ Ecological, evolutionary & environmental sciences

For a reference copy of the document with all sections, see nature.com/documents/nr-reporting-summary-flat.pdf

Life sciences study design

All studies must disclose on these points even when the disclosure is negative.

Sample size	Sample size is not applicable for this study.
Data exclusions	No data was excluded from the analysis
Replication	Functional studies: LLO elongation was performed multiple times and replication was successful.<br>Structural studies: Replication is part of the single-particle cryo-EM data processing.
Randomization	Randomization is not applicable for this study.
Blinding	Blinding is not applicable for this study.

Reporting for specific materials, systems and methods

We require information from authors about some types of materials, experimental systems and methods used in many studies. Here, indicate whether each material, system or method listed is relevant to your study. If you are not sure if a list item applies to your research, read the appropriate section before selecting a response.

Materials & experimental systems

n/a	Involved in the study	
☒	☐	Antibodies
☐	☒	Eukaryotic cell lines
☒	☐	Palaeontology
☒	☐	Animals and other organisms
☒	☐	Human research participants
☒	☐	Clinical data

Methods

n/a	Involved in the study	
☒	☐	ChIP-seq
☒	☐	Flow cytometry
☒	☐	MRI-based neuroimaging

Eukaryotic cell lines

Policy information about cell lines

Cell line source(s)	Only the following yeast strain was used in this study: Saccharomyces cerevisiae BY4742 Δost6::Leu, OST4-1D4::KanX
Authentication	The strains was authenticated by using it for OST purification.
Mycoplasma contamination	Non applicable for culture of yeast cells.
Commonly misidentified lines (See ICLAC register)	No commonly misidentified cell lines were used in this study
